# Evaluation of a class of isatinoids identified from a high-throughput screen of human kinase inhibitors as anti-Sleeping Sickness agents

**DOI:** 10.1371/journal.pntd.0007129

**Published:** 2019-02-08

**Authors:** Dana M. Klug, Rosario Diaz-Gonzalez, Guiomar Pérez-Moreno, Gloria Ceballos-Pérez, Raquel García-Hernández, Veronica Gomez-Pérez, Luis Miguel Ruiz-Pérez, Domingo I. Rojas-Barros, Francisco Gamarro, Dolores González-Pacanowska, María S. Martínez-Martínez, Pilar Manzano, Lori Ferrins, Conor R. Caffrey, Miguel Navarro, Michael P. Pollastri

**Affiliations:** 1 Northeastern University Department of Chemistry & Chemical Biology, Boston, MA, United States of America; 2 Instituto de Parasitología y Biomedicina “López-Neyra” Consejo Superior de Investigaciones Cientificas (CSIC), Granada, Spain; 3 Tres Cantos Medicines Development Campus, DDW and CIB, GlaxoSmithKline, Tres Cantos, Spain; 4 Center for Discovery and Innovation in Parasitic Diseases, Skaggs School of Pharmacy and Pharmaceutical Sciences, University of California, San Diego, La Jolla, CA, United States of America; University of Texas at El Paso, UNITED STATES

## Abstract

New treatments are needed for neglected tropical diseases (NTDs) such as Human African trypanosomiasis (HAT), Chagas disease, and schistosomiasis. Through a whole organism high-throughput screening campaign, we previously identified 797 human kinase inhibitors that grouped into 59 structural clusters and showed activity against *T*. *brucei*, the causative agent of HAT. We herein report the results of further investigation of one of these clusters consisting of substituted isatin derivatives, focusing on establishing structure-activity and -property relationship scope. We also describe their *in vitro* absorption, distribution, metabolism, and excretion (ADME) properties. For one isatin, **NEU-4391**, which offered the best activity-property profile, pharmacokinetic parameters were measured in mice.

## Author summary

Human African trypanosomiasis (HAT) is a parasitic disease prevalent in sub-Saharan Africa. Current treatments cause severe toxicity, are difficult to administer, and are susceptible to resistance. In order to quickly discover new leads for HAT drug discovery, we screened human kinase inhibitors against *Trypanosoma brucei*, the parasite that causes HAT, and discovered several hundred compounds that demonstrated antiparasitic activity. In this paper, we present the results of medicinal chemistry follow-up work on a group of compounds known as isatins.

## Introduction

Human African trypanosomiasis (HAT), also known as sleeping sickness, is a parasitic disease endemic in 36 African countries. Along with 19 other indications, HAT is designated as a neglected tropical disease (NTD) by the World Health Organization [1, 2]. Although there were fewer than 3,000 reported cases in 2015, the current burden of HAT amounts to 390,100 disability-adjusted life years [2, 3]. Caused by two subspecies of the parasite *Trypanosoma brucei* (*T*. *b*. *gambiense* and *T*. *b*. *rhodesiense*), HAT proceeds in two stages following initial infection via the bite of a tsetse fly. In the first stage, the parasite is present in the blood and lymph systems of the patient and causes mild, flu-like symptoms. In the second stage, the parasite crosses the blood-brain barrier (BBB) into the central nervous system (CNS) and causes a variety of neurological and behavioral changes, including disrupted sleeping patterns [2]. HAT is 100% fatal if left untreated, and the available drugs are associated with safety concerns, susceptibility to resistance, and lack of efficacy against all *T*. *brucei* subspecies and stages of the disease [4]. Currently, there are two new compounds for HAT in clinical trials: fexinidazole and acoziborole [5, 6]. However, given the high failure rate of compounds in clinical trials [7], it is prudent to continue to search for compounds to fill the drug discovery pipeline for HAT.

It has been shown by others that *T*. *brucei* expresses essential kinases [8], and furthermore, by our group, that human kinase inhibitors can be successfully re-optimized against these parasites [9–11]. As part of a lead repurposing strategy [12], we tested over 40,000 human kinase inhibitors in a high-throughput screen (HTS) against *T*. *b*. *brucei* [13]. This initial screening set was narrowed to 797 compounds with *T*. *b*. *brucei* pEC_50_ >6 and >100× selectivity over HepG2 cells. These final hits were then clustered based on structural similarity. We herein report the development of structure-activity and structure-property relationships (SAR and SPR) for one of these clusters.

**Fig 1:**
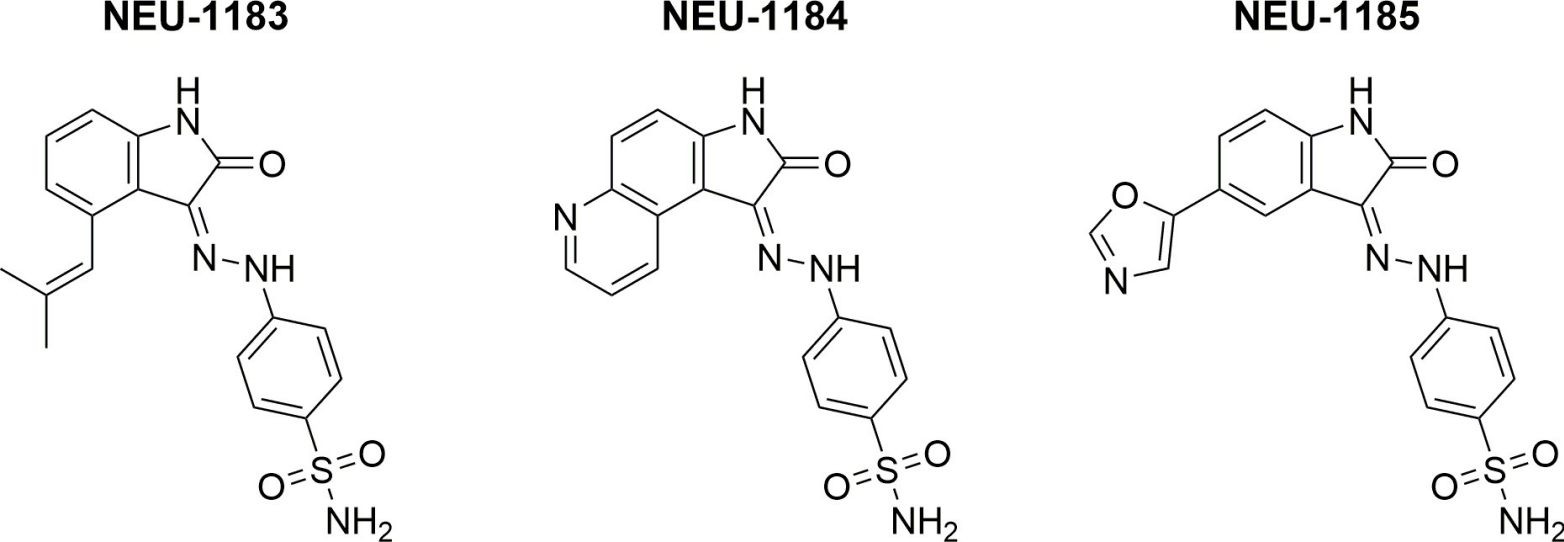
Structures of NEU-1183, -1184, and -1185.

The compounds **NEU-1183**, **NEU-1184**, and **NEU-1185** (**Fig 1**) are representatives of a cluster of isatinoids that were identified in our kinase-targeted HTS as inhibitors of *T*. *brucei* growth. Various measured and computed properties of this cluster are shown in **Table 1**, along with our targeted values for each property. In addition to physicochemical properties such as clogP (calculated partition coefficient) and topological polar surface area (TPSA), we also considered metrics such as lipophilic ligand efficiency (LLE) [14] and CNS multi-parameter optimization (CNS-MPO) scores [15] when evaluating compounds. Overall, the isatinoids had good to excellent physicochemical properties that made them an attractive starting point for further development. Their generally low clogP and high LLE values suggested that expansion of the structure would be tolerated from a property standpoint if necessary, and their high CNS-MPO scores indicated a likelihood of brain penetration (necessary for treatment of stage 2 infection). We therefore looked at ways to improve the potency and aqueous solubility of these compounds while maintaining their desirable physicochemical profile.

**Table 1:** Targeted values, cluster average, and individual values for the physicochemical properties of interest of NEU-1183, NEU-1184 and NEU-1185. Data from original HTS [13]. *nd* = no data.

	Targeted Value	Cluster Average	NEU-1183	NEU-1184	NEU-1185
***T*.*b*.*b*. pEC**_**50**_	≥7.0	7.0	7.6	7.1	6.4
**HepG2 pTC**_**50**_^a^	<5.0	4.3	5.0	4.0	4.0
**cLogP**	≤3	1.1	2.3	0.52	0.41
**LLE**	≥5	5.9	5.3	6.5	6.0
**TPSA (Å**^**2**^**)**	40<x≤90	127	114	127	140
**MWt (Da)**	≤360	374	370	367	383
**CNS-MPO Score**	≥3	4.3	4.2	4.5	4.1
**Kinetic aq. solubility (μM)**	>10	*nd*	<5	7.7	18

^a^TC_50_ = 50% toxic concentration.

## Methods

### Bioactivity assays

In order to determine the *T*. *b*. *brucei* EC_50_ values, 4 μL per well from compound master plates were dispensed into a new plate and 96 μL of HMI-9 per well were added to generate a 4% DMSO intermediate plate. Mid-log phase growth *T*. *b*. *brucei* was diluted to a working cell density of 2,750 cells/mL and 90 μL/well dispensed into 96-well flat-bottom transparent assay plates (Nunc). Ten μL/well from intermediate plates were added. The final top concentration of compounds was 40 μM in 0.4% DMSO per well.

Assay plates were incubated for 72 h at 37°C and 5% CO_2_. Four hours prior to the end of the incubation, 20 μL of a 440 μM resazurin solution in prewarmed HMI-9 was added to each well and incubated for another 4 h. Fluorescence was then measured in an Infinite F200 plate reader (Tecan) at 550 nm (excitation filter) and 590 nm (emission filter). A 4-parameter equation was employed to fit the dose-response curves and determine of EC_50_ using the SigmaPlot 13.0 software. Assays were performed in duplicate at least twice, to achieve a minimal n = 3 per dose response.

Detailed protocols for rate of action assays, *Trypanosoma cruzi* and *Leishmania donovani* EC_50_ assays, MRC5 and THP-1 cytotoxicity assays, and *Schistosoma mansoni* assays are provided in **S2 Text**.

### Pharmacokinetics protocols

**NEU-4391** was administered intraperitoneally (IP) to two groups of female NMRI mice (Group 1 n = 3; Group 2 n = 6). The compound was prepared in 1% (v/v) DMSO:99% (v/v) 20% (w/v) sulfobutyl ether-beta-cyclodextrin (SBE-β-CD) (Captisol) in water and the dosing volume was 10 mL/kg for a total dose of 10 mg/kg. Food and tap water were available *ad libitum*. Following IP dosing, Group 1 blood samples were collected from the tail vein into capillary tubes containing K2EDTA at the following time-points: 0.0833, 0.25, 0.5, 1, 2, 4, 6, 8 and 24 h.

In order to obtain simultaneous blood and brain samples, Group 2 mice were placed under terminal anaesthetic (isoflurane) and blood samples (0.3 mL) collected from the retro-orbital sinus into K2EDTA tubes at 0.5 h (n = 3) and 4 h (n = 3) after compound administration. Immediately following blood sample collection, death was confirmed by cervical dislocation and the brain removed. Aliquots of each blood sample were diluted in an equal volume of water. Mouse brain samples were weighed, water was added at a 1/2 (w/v) ratio (brain/water), and then homogenized. Both blood and brain samples were stored -80°C until analysis.

Diluted blood and brain homogenates were processed under standard liquid-liquid extraction procedures using acetonitrile containing an internal standard (Nifedipine) and analyzed by LC-MS/MS. Non-compartmental analysis was performed using the Phoenix pharmacokinetic software version 1.4 (Certara) and C_max_, t_max_, AUC_las_t, AUC, and t_1/2_ were estimated.

### Ethics statement

All animal studies were ethically reviewed and carried out in accordance with Animals (Scientific Procedures) Act 1986 and the GSK Policy on the Care, Welfare and Treatment of Animals. This work was performed at Charles River Laboratories, Edinburgh Ltd. under the UK Home Office Project Procedure Project License No. PPL 70/8781: Drug Metabolism and Pharmacokinetics, Protocol Reference Number 1 and 6.

### Chemistry

All sulfonamide-replacement analogs were synthesized by first preparing the requisite hydrazine from an aryl chloride or bromide, and subsequently coupling with isatin by stirring in methanol at room temperature. A mixture of isomers was isolated by vacuum filtration and further resolved by purification or recrystallization. All compounds tested had a purity of >95% as measured by LCMS. Details regarding compound syntheses and characterization are provided in **S1 Text**.

## Results

**Fig 2:**
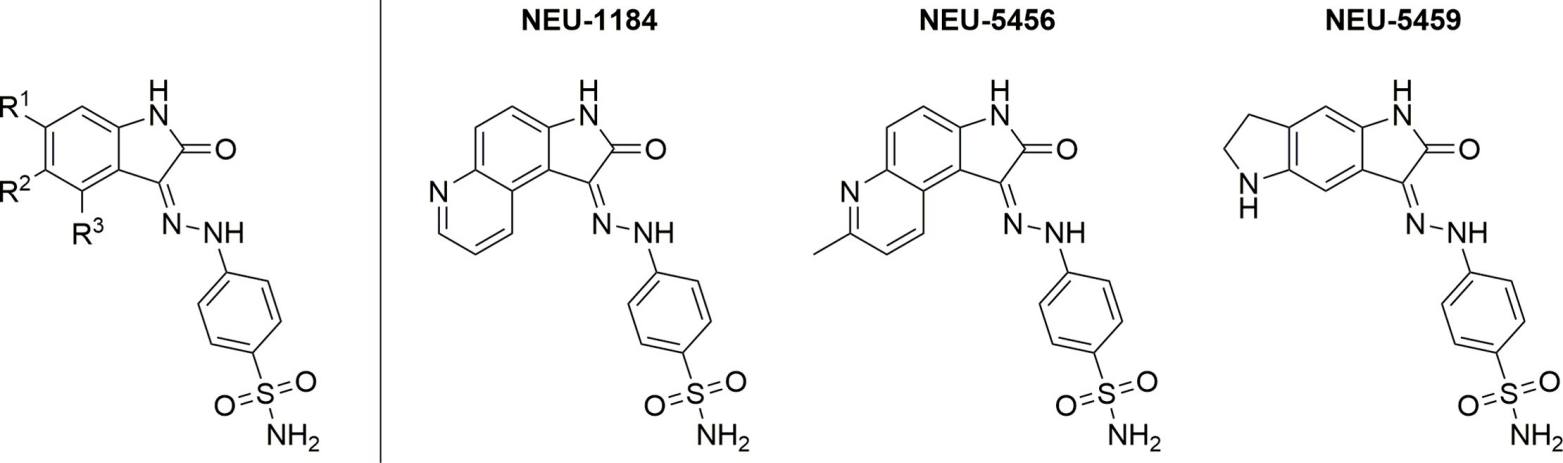
General structure of analogs presented in Table 2 as well as structures of NEU-1184, -5456, and -5459.

In addition to the three compounds shown in **Table 1**, analogs with substituents at the C4, C5, and C6 (R^3^, R^2^, and R^1^, respectively) were obtained from the GSK compound collection and tested against *T*. *brucei*; the biological activity of these compounds is presented in **Table 2** (the general structure of analogs presented is shown in **Fig 2**). The most potent of these compounds was **NEU-5469**, with methyl groups at R^1^ and R^3^ and a hydroxy group at R^2^. Changing R^3^ to a chloro, as in **NEU-5485**, resulted in a log unit drop in potency; further changing R^1^ to an isopropyl group (**NEU-5455**) resulted in another drop in potency of over half a log unit. Further exploration of the R^3^ substituent showed that in general, small alkyl substituents were best in terms of both potency and LLE. The ethyl (**NEU-5489**), isopropyl (**NEU-5491**), isobutyl (**NEU-5487**), and isobutylene (**NEU-1183**) were approximately equipotent, although **NEU-1183** has a slightly improved LLE due to its low clogP (2.2 versus **NEU-5487**’s 3.4). Amides (**NEU-5460**) and larger substituents (**NEU-5492** and **NEU-5493**) at this position generally resulted in less potent compounds as compared to the alkyl substituents. Analogs substituted at both R^2^ and R^3^ (**NEU-5464**) were significantly more potent than analogs substituted only at R^2^ (**NEU-2319**). Comparing **NEU-5464** to **NEU-5479**, the methyl substituent at R^2^ results in a loss of potency of almost half a log unit as compared to the chlorine, showing that in this case, chlorine does not act as a bioisostere for a methyl group. Aromatic fused ring systems, such as those of **NEU-1184** and **NEU-5456**, were more potent than the aliphatic ring of **NEU-5459**. As a general measure of mammalian cell toxicity, compounds were tested against either HepG2 (original hits) or MRC5 (follow-up compounds) cell lines; no analogs showed significant toxicity against either cell type.

**Table 2:** Potency, LLE, and toxicity data for substituted-core analogs.

	R^1^	R^2^	R^3^	*T*.*b*.*b*. pEC_50_	*T*.*b*.*b*. LLE	pTC_50_
NEU-1183*	-H	-H	-CHC(CH_3_)_2_	7.5	5.3	5.0^a^
NEU-1185*	-H	5-oxazole	-H	6.4	6.0	<4.0^a^
**NEU-2319**	-H	-Cl	-H	6.0	3.7	<5.0^b^
**NEU-5455**	-iPr	-OH	-Cl	6.4	3.2	<5.0^b^
**NEU-5460**	-H	-H	-CONH_2_	6.2	5.7	<5.0^b^
**NEU-5464**	-H	-Cl	-CH_3_	7.3	5.2	<5.0^b^
**NEU-5469**	-CH_3_	-OH	-CH_3_	8.2	5.8	<5.0^b^
**NEU-5470**	-Et	-H	-H	6.1	3.9	<5.0^b^
**NEU-5479**	-H	-CH_3_	-CH_3_	6.9	4.2	<5.0^b^
**NEU-5485**	-CH_3_	-OH	-Cl	7.1	4.6	<5.0^b^
**NEU-5487**	-H	-H	-CH_2_CH(CH_3_)_2_	7.4	4.0	<5.0^b^
**NEU-5489**	-H	-H	-Et	7.5	4.9	<5.0^b^
**NEU-5491**	-H	-H	-iPr	7.6	4.7	<5.0^b^
**NEU-5492**	-H	-H	-CH_2_cyBu	6.9	3.6	<5.0^b^
**NEU-5493**	-H	-H	-CH2CH2-4-hydroxybenzene	6.0	2.1	<5.1^b^
NEU-1184*	See **Fig 2**			7.1	6.4	<4.0^a^
**NEU-5456**	See **Fig 2**			7.4	5.5	<5.0^b^
**NEU-5459**	See **Fig 2**			6.1	4.9	<5.0^b^

^a^HepG2 toxicity

^b^MRC5 toxicity.

*Data from original HTS [13]. All experimental error was within ±0.20 log units.

**Fig 3:**
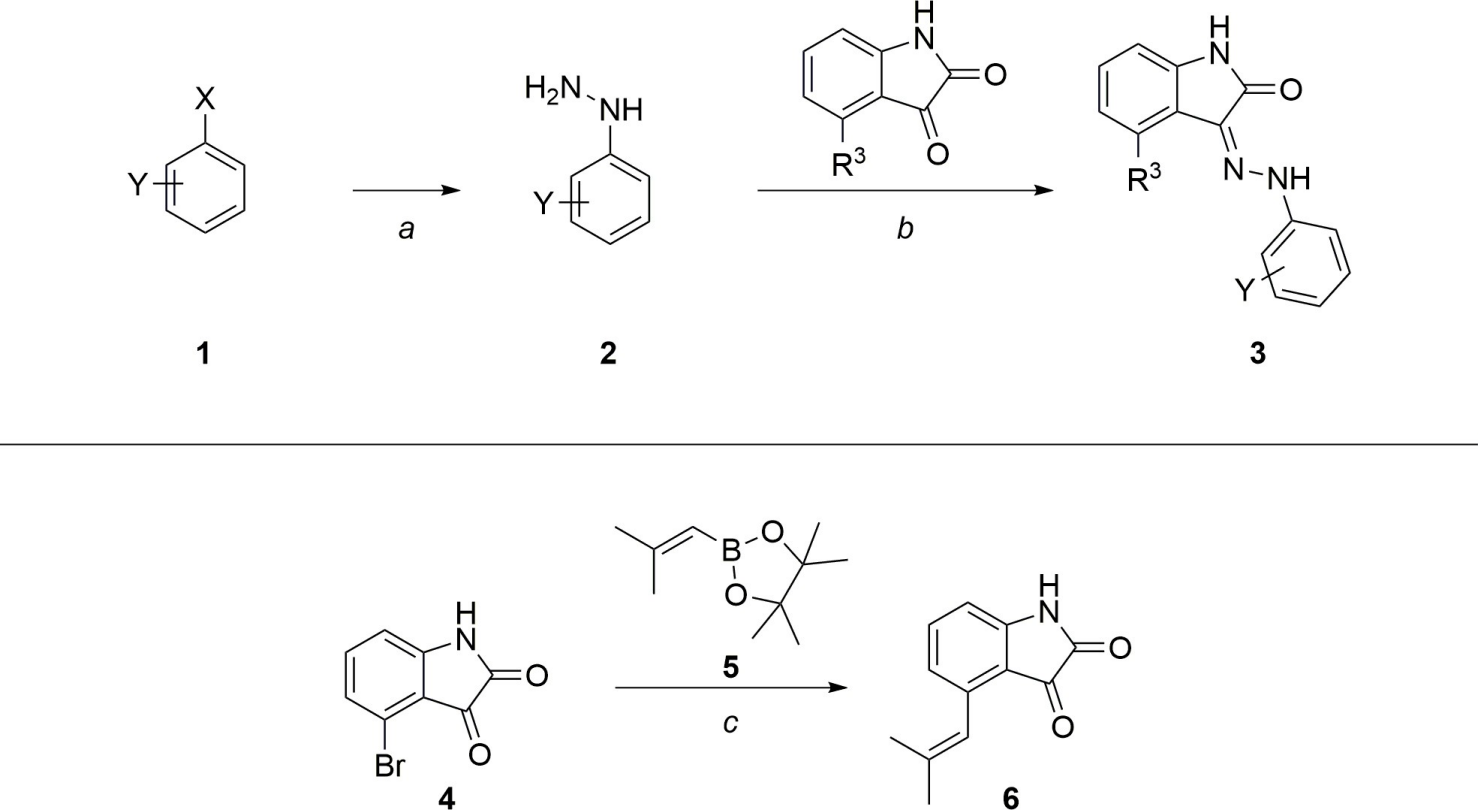
General scheme for synthesis of sulfonamide replacements and construction of substituted isatin core. Reagents and reaction conditions: a) Aryl halide, hydrazine monohydrate; 120°C, 12 h (52–86%). b) Isatin, methanol; RT, 12 h (10–100%). c) 4-Bromoisatin, boronic acid pinacol ester, K_2_CO_3_, Pd(PPh_3_)_4_; 100°C, 12 h (66%).

Given the established SAR around the isatin core, we decided to focus further efforts on replacing the sulfonamide moiety. Because of easier synthetic accessibility, initial analogs were synthesized without any substituents on the core by first making the requisite hydrazine **2** from the aryl halide **1** (X = Br or Cl) and coupling with isatin as shown in **Fig 3**. The double bond geometry of **3** was confirmed by X-ray crystallography (**S1 Fig**; CCDC ID 1865900) to be the (Z) isomer as shown in **Fig 3**, where an intramolecular hydrogen bond is formed between the amide carbonyl and the hydrazone -NH. Later, analogs with an isobutylene substituent at R^3^ were also synthesized; this core was constructed via a Suzuki coupling with 4-bromoisatin **4** and boronic ester **5** as shown in **Fig 3**.

**Fig 4:**
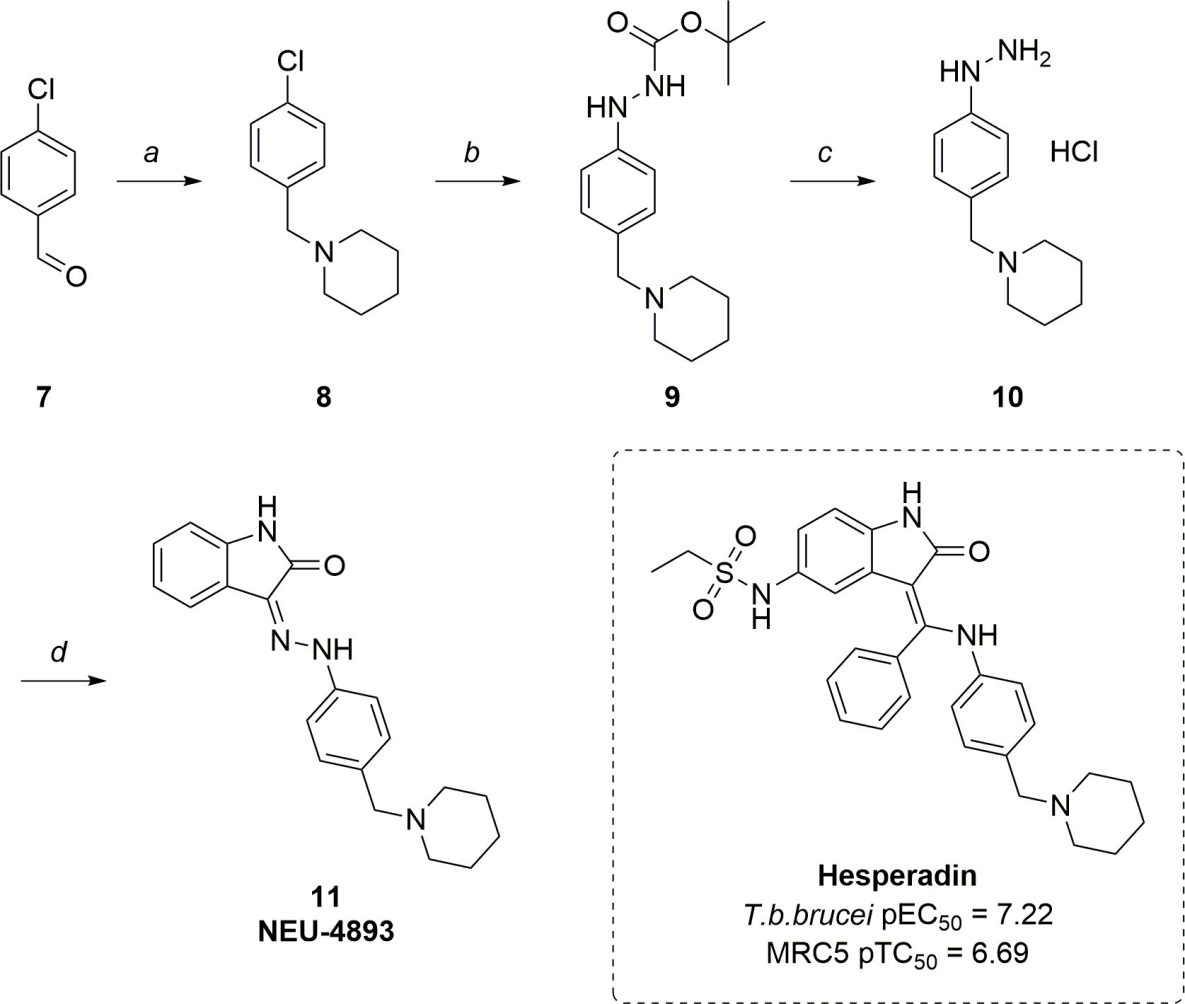
*T*. *b*. *brucei* activity of hesperadin and synthetic scheme for synthesis of NEU-4893. Reagents and reaction conditions: a) Piperidine, TEA, AcOH, NaHB(OAc)_3_, DCM; RT, 12 h (85%). b) Boc-NH-NH_2_, NaO*t*Bu, Pd_2_(dba)_3_, XPhos, dioxane; μw, 150°C, 2 h (74%). c) 4M HCl in dioxane; RT, 3 h (74%). d) NaO*t*Bu, isatin, MeOH; RT-50°C, 48 h (28%).

Additionally, we noted the structural similarity of this chemotype to hesperadin, an investigational human Aurora kinase inhibitor that we previously demonstrated as active against *T*. *brucei* (**Fig 4**) [16]. With this in mind, **NEU-4893** was designed as a crossover analog. Synthesis, shown in **Fig 4**, began with a reductive amination between 4-chlorobenzaldehyde **7** and piperidine, followed by displacement of the chloride under Buchwald conditions with boc-hydrazine to yield **9**. This compound was then deprotected using HCl to yield the HCl salt **10**, which was coupled with isatin to yield the final compound **NEU-4893**.

**Fig 5:**
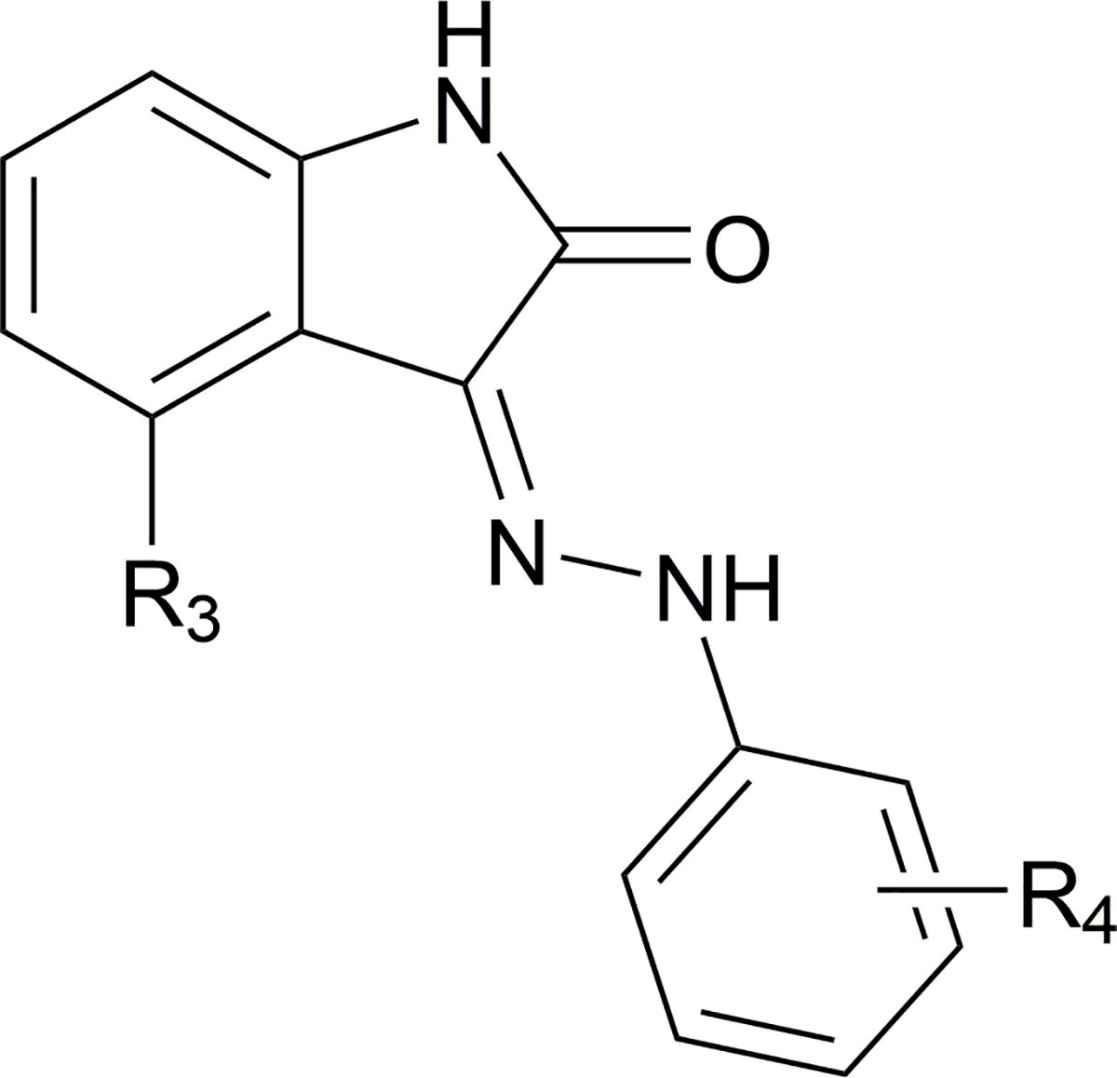
General structure of analogs presented in Table 3.

The biological activities of the sulfonamide replacement analogs are shown in **Table 3** (the general structure of analogs presented is shown in **Fig 5**). The removal of the olefin at R^3^ resulted in the loss of nearly two log units of potency against *T*. *brucei*, illustrated by **NEU-2115**. Replacement of the sulfonamide invariably resulted in a loss of potency as compared to the unsubstituted primary sulfonamide **NEU-2115**, demonstrating that this moiety is critical for activity. Tuning the electronics of the aryl ring at R^4^ had a modest effect on potency; where the electron-rich **NEU-2114** was approximately equipotent to the unsubstituted **NEU-2116**, the electron-poor **NEU-2117** lost 0.5 log units of activity compared to these two analogs. Alkylation of the primary sulfonamide resulted in a similar loss of activity, although secondary (**NEU-2124**) and tertiary (**NEU-2118**) sulfonamides were better tolerated than the excision of the sulfonamide moiety altogether. Re-introduction of the olefin substituent at R^3^ (as in **NEU-4391** and **NEU-4405**) predictably resulted in improved potency in the case of **NEU-4391**, although this came at the cost of increased clogP. Incorporation of the benzyl piperidine moiety of **NEU-4893** led to decreased activity.

**Table 3:** Potency, LLE, and toxicity data for sulfonamide replacement analogs.

	R^3^	R^4^	*T*.*b*.*b*. pEC_50_	*T*.*b*.*b*. LLE	MRC5 pTC_50_
NEU-1183*	-CHC(CH_3_)_2_	4-SO_2_NH_2_	7.6	5.3	<4.3
**NEU-2114**	H	4-OCH_3_	5.0	2.1	<4.3
**NEU-2115**	H	4-SO_2_NH_2_	5.9	4.2	<4.3
**NEU-2116**	H	n/a	4.9	1.8	<4.3
**NEU-2117**	H	3,5-dichloro	<4.4	0.12	<4.3
**NEU-2118**	H	4-SO_2_NMe_2_	5.8	3.6	<4.3
**NEU-2124**	H	4-SO_2_NHMe	5.3	3.4	<4.3
**NEU-4391**	-CHC(CH_3_)_2_	4-SO_2_NHMe	6.8	3.5	<4.3
**NEU-4405**	-CHC(CH_3_)_2_	4-SO_2_NMe_2_	5.3	1.8	<4.3
**NEU-4893**	H	4-CH_2_-piperidine	5.0	1.5	<4.3

*Data from original HTS [13]. All experimental error within ±0.08 log units.

The absorption, distribution, metabolism, and excretion (ADME) properties of the sulfonamide replacement analogs were also assessed (**S1 Table**). In general, the aqueous solubility of these compounds is low, with only two (**NEU-2124** and **NEU-4893**) achieving a solubility above 10 μM. Apart from **NEU-2116**, human liver microsome (HLM) clearance is low for this cluster. However, rat hepatocyte clearance shows significantly higher variability across the series, suggesting that mechanisms other than metabolism by CYPs may be involved in clearance. **Table 4** shows the ADME profile of two compounds of interest, **NEU-4893** and **NEU-4391**. **NEU-4893** shows the best overall ADME profile of the sulfonamide replacement analogs, but was inactive against *T*. *brucei* and for this reason was not pursued further. **NEU-4391** showed sub-micromolar activity against *T*. *brucei* and had a reasonable ADME profile, aside from high rat hepatocyte clearance.

**Table 4:** ADME profile of NEU-4893.

	Targeted Value	NEU-4893	NEU-4391
**clogP**	≤3	3.5	3.3
**LogD (7.4)**	≤2	2.2	*nd*
**Aq sol (μM)**	>10	26	4
**HLM Cl**_**int**_ **(μL/min/mg)**	<9	<3	8.6
**Rat Hepatocyte Cl**_**int**_ **(μL/min/10**^**6**^ **cells)**	<5	148	103
**PPB (%)**	≤95	90	>96

Values that meet the target are shaded green, those in an intermediate range are shaded yellow, and those that are well outside the target value are shaded red.

**Fig 6:**
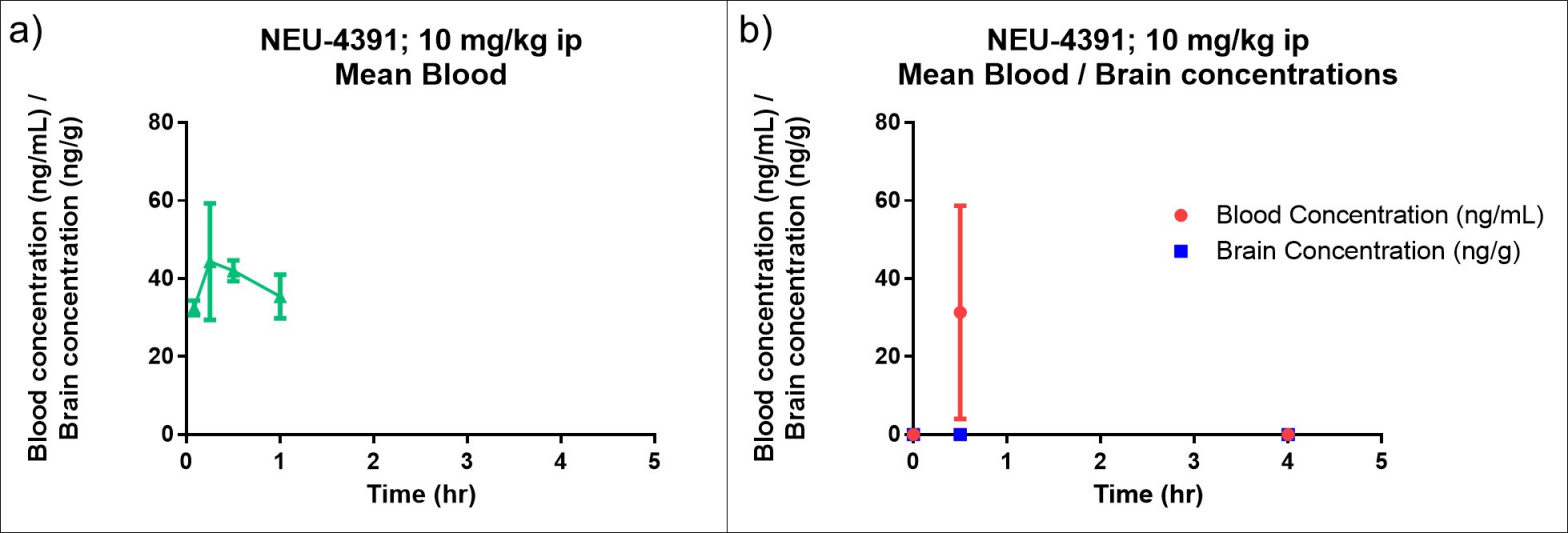
a) Peripheral blood levels of NEU-4391 after IP administration at 10 mg/kg. b) Blood and brain levels at 0. 0.5, and 4 h. Female NMRI mice (n = 3). 1% DMSO:99%, 20% Captisol in water. Individual values for each time point are represented in the plot. Concentrations after 1 h in **Fig 6A** were below the lower limit of quantitation.

Of the active compounds for which ADME data was available, **NEU-4391** displayed the best combination of potency and favorable ADME properties. We therefore progressed this compound to mouse pharmacokinetic (PK) studies using an IP dose of 10 mg/kg (**Fig 6**). The concentration of **NEU-4391** in blood is represented in **Fig 6A**, with a C_max_ of 50.1 ng/ml (0.130 μM) (**S2 Table**). This concentration is within two-fold of the EC_50_ of **NEU-4391** (0.084 μM) and is unlikely to produce a therapeutic effect *in vivo*. Additionally, the blood and brain concentrations of **NEU-4391** were measured in a separate experiment (**Fig 6B**). These results show that the concentration of **NEU-4391** in the brain was below the limit of detection for all time points (**S3 Table**). The combined PK results clearly indicate that **NEU-4391** is unlikely to be an effective therapeutic for either stage 1 or stage 2 HAT.

## Discussion

The modest potency and suboptimal ADME-PK properties suggest that this cluster of isatins is unsuitable for further development as anti-HAT agents. Although we discovered a compound, **NEU-4893**, with an improved ADME profile over the original HTS hits, this compound lost significant activity against *T*. *brucei* (approximately 2.5 log units as compared to **NEU-1183**). The compound with the best combination of potency and ADME parameters, **NEU-4391**, was progressed to *in vivo* PK studies, but both blood and brain concentrations were too low to warrant further progression.

Given our success with parasite cross-screening campaigns in the past [17], a selection of the compounds synthesized were also tested against the related parasites *T*. *cruzi*, the causative agent of Chagas disease and *L*. *donovani*, one of the causative agents of visceral leishmaniasis (**S3 Table**). We have also previously observed activity against *S*. *mansoni*, one of the causative agents of schistosomiasis, in compounds derived from other HTS clusters; we therefore tested a selection of isatin derivatives against *S*. *mansoni* (**S5 Table**). These compounds did not show activity against either *T*. *cruzi* or *L*. *donovani*, apart from the weak activity of **NEU-4391** and **NEU-1183** against *T*. *cruzi* (pEC_50_ = 5.65 and 6.14, respectively). **NEU-1183**, **-2118**, and **-2124** showed moderate and variable activity against *S*. *mansoni* adults and/or post-infective larvae (somules), but based on the overall results this cluster did not warrant further investigation as anti-schistosomal agents. We report these results in the interest of informing others who may be working on optimization programs based on our initially-reported HTS results.

## Supporting information

S1 Table ADME properties of selected analogs. (DOCX)

S2 Table Individual blood pharmacokinetic parameters of NEU-4391. (DOCX)

S3 Table Blood and brain levels of NEU-4391 after intraperitoneal administration of 10 mg/kg single dose. (DOCX)

S4 Table *T*. *cruzi* and *L*. *donovani* activity for all analogs in the isatinoid cluster. (DOCX)

S5 Table *S*. *mansoni* activity of selected analogs. (DOCX)

S1 Fig Crystal structure of NEU-2114. (TIF)

S2 Fig Rate of action curve for NEU-4391. (TIF)

S1 Text Detailed chemical synthesis and characterization. (DOCX)

S2 Text Cell assay protocols. (DOCX)

S3 Text ADME experiment protocols. (DOCX)
