# Supplementary material for: Evaluation of a class of isatinoids identified from a high-throughput screen of human kinase inhibitors as anti-Sleeping Sickness agents
Source: PLoS Negl Trop Dis. 2019 Feb 8;13(2):e0007129. doi: 10.1371/journal.pntd.0007129 (PMC6383948; doi:10.1371/journal.pntd.0007129)
Supplement: S1 Table — (DOCX) [file pntd.0007129.s001.docx]

**S1 Table.** ADME properties of selected analogs. *nd* = no data.

|  | **Aq. Solubility (µM)** | **HLM Cl_int_**  **(µL/min/mg)** | **Rat Hepatocyte Cl_int_ (µL/min/10^6^ cells)** | **PPB (%)** | **clogP** | **LogD (7.4)** |
| --- | --- | --- | --- | --- | --- | --- |
| **NEU-2114** | <7 | *nd* | *nd* | >97 | 2.9 | >3.3 |
| **NEU-2115** | <8 | 20.4 | *nd* | 96 | 1.7 | 2.3 |
| **NEU-2116** | <2 | 184 | *nd* | >98 | 3.1 | >3.8 |
| **NEU-2117** | <3 | *nd* | *nd* | >96 | 4.3 | >3.7 |
| **NEU-2118** | <1 | 38.2 | 146 | 99 | 2.1 | 3.4 |
| **NEU-2124** | <39 | 20.3 | 36.4 | 98 | 1.9 | 2.8 |
| **NEU-4391** | 4 | 8.63 | 103 | >96 | 3.3 | *nd* |
| **NEU-4405** | *nd* | *nd* | *nd* | *nd* | *nd* | *nd* |
| **NEU-4893** | 26 | <3 | 148 | 90 | 3.5 | 2.2 |
