# Supplementary material for: Evaluation of a class of isatinoids identified from a high-throughput screen of human kinase inhibitors as anti-Sleeping Sickness agents
Source: PLoS Negl Trop Dis. 2019 Feb 8;13(2):e0007129. doi: 10.1371/journal.pntd.0007129 (PMC6383948; doi:10.1371/journal.pntd.0007129)
Supplement: S2 Table — (DOCX) [file pntd.0007129.s002.docx]

**S2 Table.** Individual blood pharmacokinetic parameters of **NEU-4391** after intraperitoneal administration of 10 mg/kg single dose. Female NMRI mice (n = 3). NC = not calculated. R<0.95.

|  | **C_max_ (ng/mL)** | **t_max_ (h)** | **AUC_0-t_ (ng·h/ml)** | **AUC**  **(ng·h/ml)** | **t_1/2_ (h)** |
| --- | --- | --- | --- | --- | --- |
| **001F** | 48.9 | 0.25 | 39.3 | NC | NC |
| **002F** | 45.0 | 0.5 | 34.5 | NC | NC |
| **003F** | 56.5 | 0.5 | 39.2 | NC | NC |
| **n** | **3** | **3** | **3** | **0** | **0** |
| **Mean** | **50.1** | **0.25-0.5** | **37.7** | **-** | **-** |
| **SD** | **5.85** |  | **2.73** | **-** | **-** |
