# Supplementary material for: Evaluation of a class of isatinoids identified from a high-throughput screen of human kinase inhibitors as anti-Sleeping Sickness agents
Source: PLoS Negl Trop Dis. 2019 Feb 8;13(2):e0007129. doi: 10.1371/journal.pntd.0007129 (PMC6383948; doi:10.1371/journal.pntd.0007129)
Supplement: S3 Table — (DOCX) [file pntd.0007129.s003.docx]

**S3 Table.** Blood and brain levels of **NEU-4391** after intraperitoneal administration of 10 mg/kg single dose. Female NMRI mice (n = 3). LLOQ (lower limit of quantitation) = 25 ng/mL (blood); 25 ng/g (brain).

| **Sampling Time (hr)** | **Matrix** | **N1** | **N2** | **N3** | **Mean** | **SD** |
| --- | --- | --- | --- | --- | --- | --- |
| **0.5** | Blood | 50.7 | <LLOQ | 43.3 | 47.0 | 5.23 |
|  | Brain | <LLOQ | <LLOQ | <LLOQ | - | - |
|  | **Brain/Blood Ratio** | **-** | **-** | **-** | **-** | **-** |
| **4.0** | Blood | <LLOQ | <LLOQ | <LLOQ | - | - |
|  | Brain | <LLOQ | <LLOQ | <LLOQ | - | - |
|  | **Brain/Blood Ratio** | **-** | **-** | **-** | **-** | **-** |
