# Supplementary material for: Evaluation of a class of isatinoids identified from a high-throughput screen of human kinase inhibitors as anti-Sleeping Sickness agents
Source: PLoS Negl Trop Dis. 2019 Feb 8;13(2):e0007129. doi: 10.1371/journal.pntd.0007129 (PMC6383948; doi:10.1371/journal.pntd.0007129)
Supplement: S4 Table — (DOCX) [file pntd.0007129.s004.docx]

**S4 Table.** *T. cruzi* and *L. donovani* activity for selected analogs in the isatinoid cluster.

|  | ***T. cruzi* pEC_50_** | ***T. cruzi* LLE** | ***L. donovani* pEC_50_** | ***L. donovani* LLE** |
| --- | --- | --- | --- | --- |
| **NEU-1183** | 6.14 ± 0.026 | 3.10 | <5.30 | 2.26 |
| **NEU-2114** | <5.00 | 2.09 | <5.00 | 2.09 |
| **NEU-2115** | <5.00 | 3.33 | <5.00 | 3.33 |
| **NEU-2116** | <5.00 | 1.93 | <5.00 | 1.93 |
| **NEU-2117** | <5.00 | 0.73 | <5.00 | 0.73 |
| **NEU-2118** | <5.00 | 2.88 | <5.00 | 2.88 |
| **NEU-2124** | <5.00 | 3.10 | <5.00 | 3.10 |
| **NEU-4391** | 5.65 ± 0.037 | 2.38 | <5.30 | 2.04 |
| **NEU-4405** | <4.70 | 1.21 | <5.30 | 1.81 |
| **NEU-4893** | <4.70 | 1.22 | <5.30 | 1.82 |
