# Supplementary material for: Evaluation of a class of isatinoids identified from a high-throughput screen of human kinase inhibitors as anti-Sleeping Sickness agents
Source: PLoS Negl Trop Dis. 2019 Feb 8;13(2):e0007129. doi: 10.1371/journal.pntd.0007129 (PMC6383948; doi:10.1371/journal.pntd.0007129)
Supplement: S5 Table — (DOCX) [file pntd.0007129.s005.docx]

**Table S5.** Activity of selected analogs against *S. mansoni* adults and somules.

|  | **Adult severity score (10 μM)** | | | | **Somule severity score (10 μM)** | |
| --- | --- | --- | --- | --- | --- | --- |
| **NEU-** | **3h** | **6h** | **24h** | **48h** | **24h** | **48h** |
| **1183** | 2 | 2 | 2 | 2 | 1 | 4 |
| **2114** | 0 | 0 | 0 | 0 | 2 | 0 |
| **2116** | 0 | 0 | 0 | 1 | 0 | 0 |
| **2117** | 1 | 0 | 0 | 3 | 0 | 0 |
| **2118** | 3 | 3 | 3 | 3 | 0 | 0 |
| **2124** | 2 | 2 | 2 | 2 | 1 | 0 |
