# Supplementary figures and images for: Evaluation of a class of isatinoids identified from a high-throughput screen of human kinase inhibitors as anti-Sleeping Sickness agents
Source: PLoS Negl Trop Dis. 2019 Feb 8;13(2):e0007129. doi: 10.1371/journal.pntd.0007129 (PMC6383948; doi:10.1371/journal.pntd.0007129)

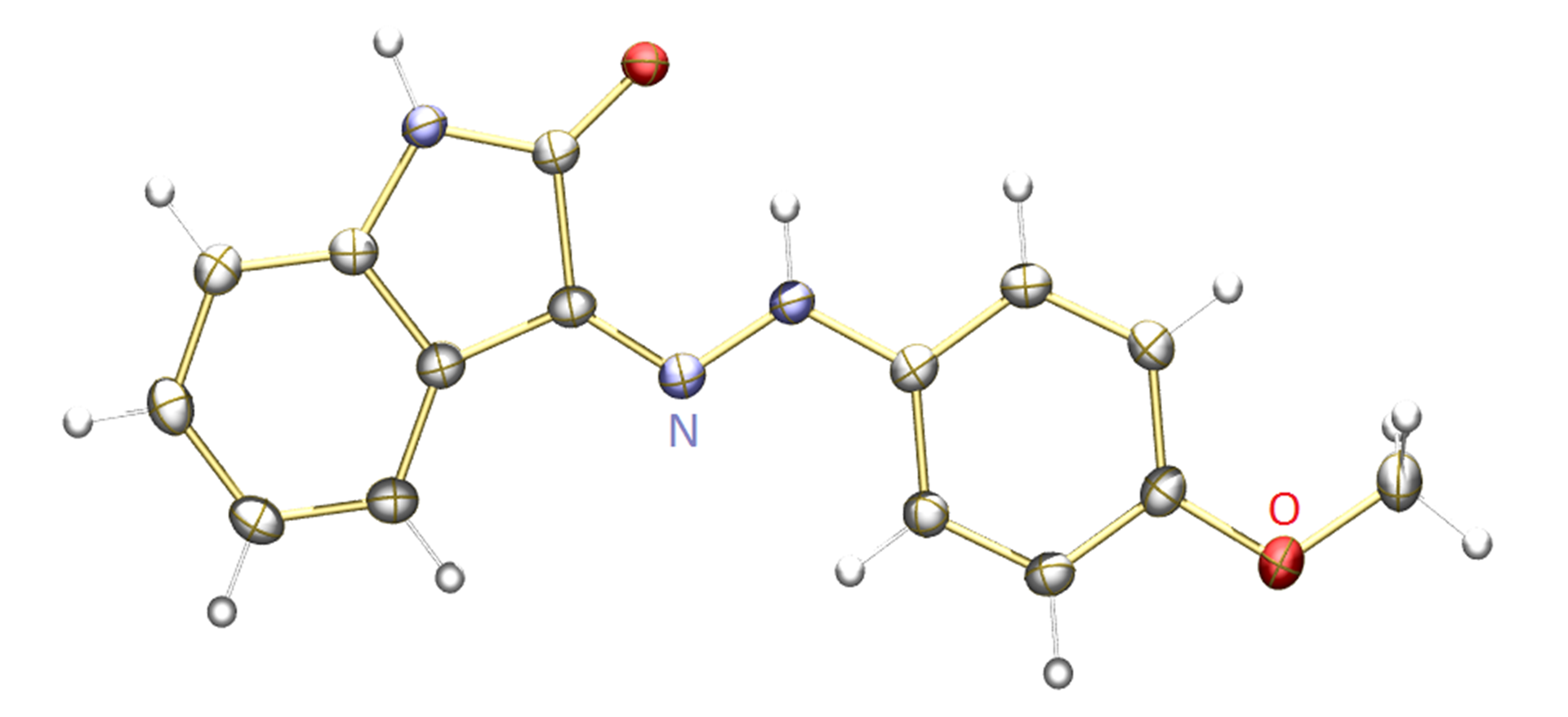

Supplement: S1 Fig — (TIF) [file pntd.0007129.s006.tif]

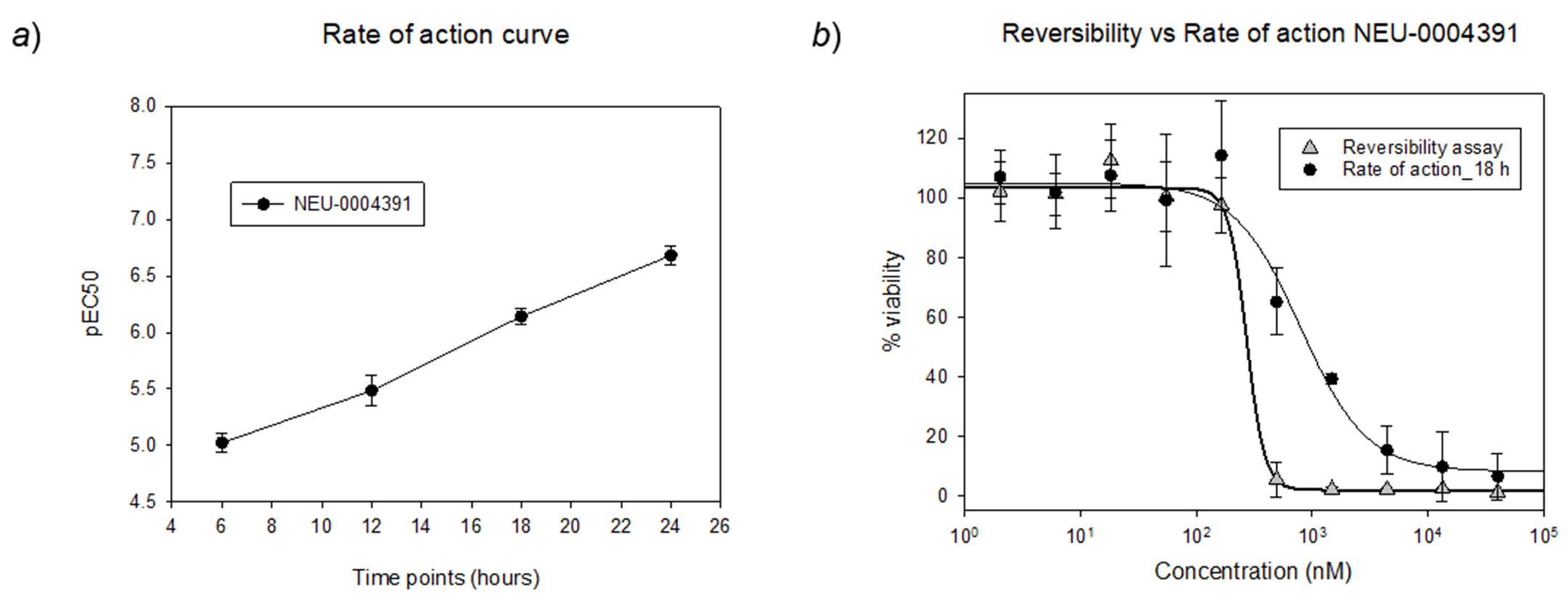

Supplement: S2 Fig — (TIF) [file pntd.0007129.s007.tif]
