# Supplementary material for: Evaluation of a class of isatinoids identified from a high-throughput screen of human kinase inhibitors as anti-Sleeping Sickness agents
Source: PLoS Negl Trop Dis. 2019 Feb 8;13(2):e0007129. doi: 10.1371/journal.pntd.0007129 (PMC6383948; doi:10.1371/journal.pntd.0007129)
Supplement: S1 Text — (DOCX) [file pntd.0007129.s008.docx]

**S1 Text.** Detailed chemical synthesis and characterization.

All compounds tested had a purity of >95% as measured by LCMS, unless otherwise noted.

Reagents purchased were used as received, unless otherwise noted. Purification of intermediates was performed using silica gel chromatography using the Biotage® Isolera™One flash purification system. LCMS analysis was performed using a Waters Alliance reverse phase HPLC using a multi-wavelength photodiode array detector from 210 nm to 600 nm.

Preparative HPLC was conducted for final compounds on Waters FractionLynx system using acetonitrile/water and 0.1% formic acid gradient and collected based on UV monitoring at 254 nm.

^1^H NMR spectra were obtained with Varian NMR systems, operating at either 400 or 500 MHz at room temperature, using solvents from Cambridge Isotope Laboratories. Chemical shifts (δ, ppm) are reported relative to the solvent peak (CDCl_3_: 7.26 [^1^H]; or DMSO-*d*_6_: 2.50 [^1^H]). Data for ^1^H NMR spectra are reported as follows: chemical shift (ppm), multiplicity (s for singlet, d for doublet, t for triplet, dd for doublet of doublet, m for multiplet), coupling constant (Hz), and integration.

**General Procedure A (For synthesis of (phenylhydrazino)indolin-2-ones)**

To a solution of the substituted indoline-2,3-dione (1 equiv.) in methanol (0.2 M) was added the substituted phenylhydrazine (2 equiv.) and the reaction was stirred overnight at room temperature. A mixture of isomers was isolated by vacuum filtration and further resolved by the stated method.

*(Z)-3-(2-(4-Methoxyphenyl)hydrazono)indolin-2-one* **NEU-2114**

The title compound was prepared according to **General Procedure A** on a 100-mg scale using indoline-2,3-dione and (4-methoxyphenyl)hydrazine. The crude material was dissolved in hot 1:1 methanol:water and then cooled to room temperature. An orange solid was isolated by vacuum filtration to afford the title compound (54 mg, 30%). LCMS [M+H]^+^ 268.1 m/z; ^1^H NMR (500 MHz, DMSO-*d*_6_) δ ppm 12.77 (s, 1H) 10.98 (s, 1H) 7.52 (d, *J* = 7.32 Hz, 1H) 7.37 - 7.41 (m, 2H) 7.22 (td, *J* = 7.30, 1.00 Hz, 1H) 7.04 (td, *J* = 7.57, 0.98 Hz, 1H) 6.95 - 6.99 (m, 2H) 6.91 (d, *J* = 7.81 Hz, 1H) 3.75 (s, 3H).

*(Z)-4-(2-(2-Oxoindolin-3-ylidene)hydrazinyl)benzenesulfonamide* **NEU-2115**

The title compound was prepared according to **General Procedure A** on a 79-mg scale using indoline-2,3-dione and 4-hydrazinylbenzenesulfonamide. The crude material was dissolved in hot 1:1 methanol:hexanes and then cooled to room temperature. A bright yellow solid was isolated by vacuum filtration to afford the title compound (152 mg, 89%). LCMS [M+H]^+^ 317.0 m/z; ^1^H NMR (500 MHz, DMSO-*d*_6_) δ ppm 12.80 (s, 1H) 11.08 - 11.15 (m, 1H) 7.77 - 7.81 (m, 2H) 7.55 - 7.61 (m, 3H) 7.25 - 7.31 (m, 3H) 7.07 (t, *J* = 7.32 Hz, 1 H) 6.93 (d, *J* = 7.81 Hz, 1H).

*(Z)-3-(2-Phenylhydrazono)indolin-2-one* **2116**

The title compound was prepared according to **General Procedure A** on a 75-mg scale using indoline-2,3-dione and phenylhydrazine. The crude material was dissolved in hot 1:1 methanol:hexanes and then cooled to room temperature. A bright yellow solid was isolated by vacuum filtration to afford the title compound (152 mg, 89%). LCMS [M+H]^+^ 238.0 m/z; ^1^H NMR (500 MHz, DMSO-*d*_6_) δ ppm 12.74 (s, 1H) 11.04 (br. s., 1H) 7.55 (d, *J* = 7.32 Hz, 1H) 7.42 - 7.45 (m, 2H) 7.35 - 7.40 (m, 2H) 7.25 (td, *J* = 7.30, 1.00 Hz, 1H) 7.02 - 7.08 (m, 2H) 6.92 (d, *J* = 7.81 Hz, 1H).

*(Z)-3-(2-(3,5-Dichlorophenyl)hydrazono)indolin-2-one* **NEU-2117**

The title compound was prepared according to **General Procedure A** on a 100-mg scale using indoline-2,3-dione and (3,5-dichlorophenyl)hydrazine hydrochloride. The crude material was dissolved in hot methanol and then cooled to room temperature. A bright yellow solid was isolated by vacuum filtration to afford the title compound (189 mg, 91%). LCMS [M+H]^+^ 306.0 m/z (^35^Cl), 308.0 m/z (^37^Cl); ^1^H NMR (400 MHz, DMSO-*d*_6_) δ ppm 12.60 (br s, 1H), 11.08 (br s, 1H), 7.64 (d, *J* = 7.52 Hz, 1H), 7.55 (d, *J* = 2.05 Hz, 2H), 7.28 (d, *J* = 7.52 Hz, 1H), 7.18 (t, *J* = 1.71, 1H), 7.06 (t, *J* = 7.52, 1H), 6.92 (d, *J* = 8.21 Hz, 1H).

*(Z)-*N*,*N*-Dimethyl-4-(2-(2-oxoindolin-3-ylidene)hydrazinyl)benzenesulfonamide* **NEU-2118**

The title compound was prepared according to **General Procedure A** on a 35-mg scale using indoline-2,3-dione and 4-hydrazinyl-*N*,*N*-dimethylbenzenesulfonamide. The title compound was isolated as a single isomer by vacuum filtration (65 mg, 79%). LCMS [M+H]^+^ 345.0 m/z; ^1^H NMR (500 MHz, DMSO-*d*_6_) δ ppm 12.82 (s, 1H) 11.14 (s, 1H) 7.69 - 7.74 (m, 2H) 7.63 - 7.66 (m, 2H) 7.59 (d, *J* = 7.81 Hz, 1H) 7.27 - 7.32 (m, 1H) 7.08 (d, *J* = 0.98 Hz, 1H) 6.94 (d, *J* = 7.81 Hz, 1H) 2.59 (s, 6H).

*(Z)-N-Methyl-4-(2-(2-oxoindolin-3-ylidene)hydrazinyl)benzenesulfonamide* **NEU-2124**

The title compound was prepared according to **General Procedure A** on a 36-mg scale using indoline-2,3-dione and 4-hydrazinyl-N-methylbenzenesulfonamide. The title compound was recrystallized using a mixture of 1:1 water:methanol (81 mg, 100%). LCMS [M+H]^+^ 330.9 m/z; ^1^H NMR (500 MHz, DMSO-*d*_6_) δ ppm 12.80 (s, 1H) 11.12 (br. s, 1H) 7.74 (d, *J* = 8.79 Hz, 2H) 7.57 - 7.63 (m, 3H) 7.33 (q, *J* = 5.37 Hz, 1H) 7.29 (td, *J* = 7.30, 1.00 Hz, 1H) 7.08 (t, *J* = 7.32 Hz, 1H) 6.93 (d, *J* = 7.81 Hz, 1H) 2.40 (d, *J* = 5.37 Hz, 3H).

*4-(2-Methylprop-1-en-1-yl)indoline-2,3-dione*

4-Bromoindoline-2,3-dione (299 mg, 1.32 mmol), and potassium carbonate (644 mg, 4.66 mmol) were suspended in 3:1 dioxane:water (16 ml, 0.08M) and 4,4,5,5-tetramethyl-2-(2-methylprop-1-en-1-yl)-1,3,2-dioxaborolane (0.400 ml, 1.22 mmol) was added. The reaction flask was degassed for ~10 minutes. Palladium tetrakis (76 mg, 65.77 µmol) was added and the reaction was run under nitrogen at 100 °C overnight, then cooled to room temperature, diluted with ethyl acetate, and filtered through celite. The crude mixture was purified by flash chromatography (0-50% ethyl acetate:hexanes) to afford the title compound as a bright orange solid (176 mg, 66%). LCMS [M+H]^+^ 202.0 m/z; ^1^H NMR (500 MHz, DMSO-*d*_6_) δ ppm 11.02 (s, 1H), 7.50 (t, *J* = 7.81 Hz, 1H), 6.98 (d, *J* = 7.81 Hz, 1H), 6.73 (d, *J* = 7.81 Hz, 1H), 6.70 (s, 1H), 1.07 (s, 6H).

*(Z)-*N*-Methyl-4-(2-(4-(2-methylprop-1-en-1-yl)-2-oxoindolin-3-ylidene)hydrazinyl)benzenesulfonamide* **NEU-4391**

The title compound was prepared according to **General Procedure A** on a 17-mg scale using 4-(2-methylprop-1-en-1-yl)indoline-2,3-dione and 4-chloro-*N*-methylbenzenesulfonamide. The mixture of isomers was resolved by recrystallization in methanol to afford the title compound as a yellow solid (18 mg, 56%). LCMS [M+H]^+^ 385.0 m/z; ^1^H NMR (500 MHz, DMSO-*d*_6_) δ ppm 12.94 (s, 1H), 11.12 (s, 1H), 7.75 (d, *J* = 8.79 Hz, 2H), 7.52 (d, *J* = 8.79, 2H), 7.29 (q, *J* = 4.90, 1H), 7.25 (t, *J* = 7.81, 1H), 6.96 (d, *J* = 8.30 Hz, 1H), 6.78 (m, 2H), 2.40 (d, *J* = 4.88 Hz, 3H), 2.05 (s, 1H), 1.85 (s, 1H).

*(Z)-*N*,*N*-Dimethyl-4-(2-(4-(2-methylprop-1-en-1-yl)-2-oxoindolin-3-ylidene)hydrazinyl)benzenesulfonamide* **NEU-4405**

The title compound was prepared according to **General Procedure A** on a 16-mg scale using 4-(2-methylprop-1-en-1-yl)indoline-2,3-dione and 4-chloro-N,N-dimethylbenzenesulfonamide. The reaction was concentrated under reduced pressure and the residue was purified by flash chromatography (20-50% ethyl acetate:hexanes), then further purified by prep HPLC (30-95% acetonitrile:water) to afford the title compound as a bright yellow solid (8 mg, 10%). LCMS [M+H]^+^ 399.0 m/z; ^1^H NMR (500 MHz, DMSO-*d*_6_) δ ppm 12.96 (s, 1H), 11.14 (s, 1H), 7.73 (d, *J* = 8.79 Hz, 2H), 7.56 (d, *J* = 8.79 Hz, 2H), 7.25 (t, *J* = 7.81 Hz, 1H), 6.96 (d, *J* = 7.81 Hz, 1H), 6.80 (d, *J* = 7.32 Hz, 1 H), 6.77 (s, 1H), 2.60 (s, 6H), 2.05 (s, 3H), 1.85 (s, 3H).

*1-(4-Chlorobenzyl)piperidine*

To a solution of 4-chlorobenzaldehyde (502 mg, 3.57 mmol) in dichloromethane (10.0 ml, 0.36 M) was added piperidine (0.9 ml, 9.11 mmol), triethylamine (0.75 ml, 5.38 mmol), and acetic acid (0.3 ml, 5.25 mmol). After stirring at room temperature for 30 minutes, NaHB(OAc)_3_ (1.90 g, 8.96 mmol) was added and the reaction continued stirring overnight. The reaction was diluted with dichloromethane and washed twice with 3M aqueous NaOH, once with water, and once with brine. The organic layer was dried with sodium sulfate and the title compound was isolated by flash chromatography (0-50% ethyl acetate:hexanes) as a colorless oil (635 mg, 85%). LCMS [M+H]^+^ 210.1 m/z (^35^Cl), 212.1 m/z (^37^Cl); ^1^H NMR (500 MHz, DMSO-*d*_6_) δ ppm 7.35 (d, *J* = 7.81 Hz, 2H) 7.30 (d, *J* = 8.30 Hz, 2H) 3.39 (s, 2H) 2.29 (br. s., 4H) 1.47 (quin, *J* = 5.37 Hz, 4H) 1.33 - 1.41 (m, 2H).

Tert*-butyl 2-(4-(piperidin-1-ylmethyl)phenyl)hydrazine-1-carboxylate*

1-(4-Chlorobenzyl)piperidine (635 mg, 3.03 mmol) was combined with tert-butyl hydrazinecarboxylate (801 mg, 6.06 mmol), sodium *tert*-butoxide (582 mg, 6.06 mmol), Pd_2_(dba)_3_ (283 mg, 0.309 mol), XPhos (144 mg, 0.302 mmol) and dry dioxane (12 ml, 0.25 M) in a microwave vial that was degassed for ~10 mins. The reaction was run in the microwave (150 °C, N abs) for 2 h. The reaction mixture was diluted with ethyl acetate and filtered through celite. The title compound was isolated as a mixture with an unknown side product by flash chromatography (20-100% ethyl acetate:hexanes – 0-15% methanol:dichloromethane) as an orange oil which was carried forward without further purification (680 mg, 74%). LCMS [M+H]^+^ 306.2 m/z.

*1-(4-Hydrazineylbenzyl)piperidine*

*Tert*-butyl 2-(4-(piperidin-1-ylmethyl)phenyl)hydrazine-1-carboxylate (680 mg, 2.23 mmol) was taken up in 4M aqueous HCl in dioxane (3.0 ml, 12.00 mmol) and the reaction was stirred at room temperature for ~3 hr. Upon completion, the reaction was concentrated under reduced pressure. The resultant red-orange solid was triturated with ethyl acetate, then with acetone. The desired product was isolated as the HCl salt and used in subsequent reactions without further purification (400 mg, 74%). LCMS [M+H]^+^ 206.2 m/z; ^1^H NMR (500 MHz, DMSO-*d*_6_) δ ppm 10.50 (br. s, 1H) 10.36 (br. s., 2H) 7.49 (d, *J* = 8.79 Hz, 2H) 7.00 (d, *J* = 8.79 Hz, 2H) 4.14 (d, *J* = 5.37 Hz, 2H) 3.22 (d, *J* = 11.72 Hz, 2 H) 2.71 - 2.84 (m, 2 H) 1.76 (m, *J*=3.40 Hz, 4 H) 1.68 (m, *J*=13.20 Hz, 2 H).

*(Z)-3-(2-(4-((Piperidin-1-ylmethyl)sulfonyl)phenyl)hydrazineylidene)indolin-2-one* **NEU-4893**

Sodium *tert*-butoxide (41 mg, 0.427 mmol) was added to a suspension of 1-(4-hydrazinylbenzyl)piperidine hydrochloride (66 mg, 0.273 mmol) in methanol (0.4 ml, 0.68 M). The reaction was stirred at room temperature for 10 minutes. A suspension of indoline-2,3-dione (20 mg, 0.136 mmol) in methanol (0.3 ml, 0.45 M) was then added and the reaction was stirred overnight at room temperature. Due to incomplete conversion, the reaction temperature was increased to 50 °C and the reaction continued to stir for another 24 h. The title compound was then isolated by vacuum filtration as a yellow solid (13 mg, 28%). LCMS [M+H]^+^ 335.2 m/z; ^1^H NMR (500 MHz, DMSO-*d*_6_) δ ppm 12.73 (br. s, 1H) 11.06 (br. s, 1H) 7.53 (d, *J* = 7.32 Hz, 1H) 7.37 (d, *J* = 8.30 Hz, 2H) 7.27 (d, *J* = 8.30 Hz, 2H) 7.24 (td, *J* = 7.57, 0.98 Hz, 1H) 7.04 (t, *J* = 7.32 Hz, 1H) 6.93 (d, *J* = 7.81 Hz, 1H) 3.38 (s, 2H) 2.30 (br. s., 4H) 1.48 (quin, *J* = 5.49 Hz, 4H) 1.38 (br. s, 2H).

*4-Hydrazinylbenzenesulfonamide*

Hydrazine monohydrate (1.3 ml, 26.5 mmol) was added to 4-chlorobenzenesulfonamide (503 mg, 2.62 mmol) and the reaction was refluxed at 120 °C for 36 h. Upon completion, the reaction mixture was cooled to room temperature. Cold (0 °C) water was added and the resulting precipitate was isolated by vacuum filtration to afford the title compound as a white solid (330 mg, 67%). LCMS [M+H]^+^ 188.8 m/z; ^1^H NMR (500 MHz, DMSO-*d*_6_) δ ppm 7.50 (d, *J* = 9.28 Hz, 2 H) 7.43 (s, 1 H) 6.93 (br. s., 1 H) 6.78 (d, *J* = 8.79 Hz, 2 H) 4.16 (br. s., 2 H).

*4-Chloro-*N*,*N*-dimethylbenzenesulfonamide*

4-Chlorobenzenesulfonamide (200 mg, 1.04 mmol) and sodium hydride (60% wt suspension in oil) (100 mg, 2.50 mmol) were suspended in anhydrous DMF (7.0 ml, 0.15 M). Iodomethane (0.2 ml, 3.20 mmol) was added dropwise and the reaction was stirred at room temperature for 2 h. The reaction mixture was diluted with ethyl acetate and washed three times with water and once with brine. The organic layer was dried with sodium sulfate and concentrated under reduced pressure. The title compound was isolated as a pale yellow solid (197 mg, 86%). LCMS [M+H]^+^ 219.9 m/z (^35^Cl), 221.8 m/z (^37^Cl); ^1^H NMR (400 MHz, CDCl_3_) δ ppm 7.73 (d, *J* = Hz, 2H), 7.53 (d, *J* = Hz, 2H), 2.72 (s, 6H).

*4-Hydrazinyl-*N*,*N*-dimethylbenzenesulfonamide*

Hydrazine monohydrate (0.5 ml, 10.20 mmol) was added to 4-chloro-*N*,*N*-dimethylbenzenesulfonamide (197 mg, 0.897 mmol) and the reaction was refluxed at 120 °C for 60 h. Upon completion, the reaction mixture was cooled to room temperature. Cold (0 °C) water was added and the resulting precipitate was isolated by vacuum filtration to afford the title compound as a white solid (100 mg, 52%). LCMS [M+H]+ 215.9 m/z; 1H NMR (400 MHz, CDCl_3_) δ ppm 7.63 (d, *J* = Hz, 2H), 6.90 (d, *J* = Hz, 2H), 5.62 (br. s, 1H), 3.68 (br. s, 2H), 2.67 (s, 6H).

*4-Bromo-*N*-methylbenzenesulfonamide*

4-Bromobenzenesulfonyl chloride (1.00 g, 3.92 mmol) and methylamine hydrochloride (1.32 g, 19.6 mmol) were dissolved in pyridine (13 ml, 0.30 M), followed by the addition of 4-dimethylaminopyridine (26 mg, 0.213 mmol). The reaction was refluxed at 100 °C for 1.5 h, after which the reaction was cooled to room temperature. The reaction mixture was then diluted with ethyl acetate and washed once with 1M HCl, once with water, and once with brine. The organic layer was dried with sodium sulfate concentrated under reduced pressure, yielding the title compound as a yellow oil (721 mg, 74%). LCMS [M+H]^+^ 249.8 (^79^Br) m/z, 251.9 (^81^Br) m/z; ^1^H NMR (400 MHz, CDCl_3_) δ ppm 7.72 - 7.76 (m, 2H) 7.65 - 7.69 (m, 2H) 4.70 (d, *J* = 5.13 Hz, 1H) 2.67 (d, *J* = 5.86 Hz, 3H).

*4-Hydrazinyl-*N*-methylbenzenesulfonamide*

Hydrazine monohydrate (2.20 ml, 44.9 mmol) was added to 4-bromo-*N*-methylbenzenesulfonamide (721 mg, 2.88 mmol) and the reaction was stirred overnight at 120 °C. Upon completion, the reaction mixture was cooled to room temperature. Cold (0 °C) water was added and the resulting precipitate was isolated by vacuum filtration to afford the title compound as a white solid (430 mg, 71%). LCMS [M+H]^+^ 201.9 m/z; ^1^H NMR (500 MHz, DMSO-*d*_6_) δ ppm 7.54 (s, 1 H) 7.45 (d, *J* = 8.79 Hz, 2 H) 6.95 (q, *J* = 5.40 Hz, 1 H) 6.80 (d, *J* = 8.79 Hz, 2 H) 4.19 (br. s., 2 H) 2.32 (d, *J* = 5.37 Hz, 3 H).
