# Supplementary material for: Evaluation of a class of isatinoids identified from a high-throughput screen of human kinase inhibitors as anti-Sleeping Sickness agents
Source: PLoS Negl Trop Dis. 2019 Feb 8;13(2):e0007129. doi: 10.1371/journal.pntd.0007129 (PMC6383948; doi:10.1371/journal.pntd.0007129)
Supplement: S2 Text — (DOCX) [file pntd.0007129.s009.docx]

**S2 Text.** Cell assay protocols.

*Strains and media*

Bloodstream *Trypanosoma brucei brucei* Lister 427 was cultured in Hirumi’s modified Iscove’s medium (HMI-9), supplemented with 10% heat-inactivated FBS, at 37 ºC and 5% CO_2_ in T-25 vented flask (Corning®).

MRC5-SV2 cell line (SV40-transformed human lung fibroblast cell line) was cultured in DMEM medium supplemented with 10% FBS at 37 ºC and 5% CO_2_ in T-75 vented flask (Corning®).

The *T. cruzi* Tulahuen C4 strain, expressing the β-galactosidase gene (LacZ) and L6 rat skeletal muscle cells, used as host cells, were cultured in RPMI-1640 supplemented with 10% iFBS, 2 mM L-glutamine, 100 U/mL penicillin, and 100 μg/mL streptomycin at 37 °C and 5% CO_2_.

*Leishmania donovani* MHOM/ET/67/HU3 cells with the luciferase gene integrated into the parasite genome[[1](#_ENREF_1)] were grown at 28 °C in RPMI 1640-modified medium (Invitrogen) supplemented with 20% FBS with 100 mg/ml of hygromycin B.

Maintenance of the *Schistosoma mansoni* life cycle, preparation of somules (post-infective larvae) and adult worms (≥42-days-old), and co-incubation of these stages with test compounds were as described. [[2-4](#_ENREF_2)] An NMRI isolate of *S. mansoni* was used for all whole-organism screens.

The Human myelomonocytic cell line THP-1 was grown at 37 °C and 5% CO_2_ in RPMI-1640 supplemented with 10% iFBS, 2 mM glutamate, 100 U/mL penicillin and 100 mg/mL streptomycin. 3 x 10^4^ THP-1 cells per well in 96-well plates were differentiated to macrophages with 20 ng/mL of PMA treatment for 48 h followed by 24 h of culture in fresh medium.

*Preparation of compound plates*

For dose-response experiments, compound plates were prepared for each analogue by serial 3-fold dilutions in 100% DMSO. Five concentration points (mammalian cytotoxicity) or ten concentration points (parasite growth inhibition), were made in 96-well transparent Nunclon plates. Pentamidine is routinely included in compound plates as internal quality control, and plates are stored sealed at -20 ºC for no more than four weeks.

*Rate of Action assays*

Mid-log *T. brucei brucei* cultures were diluted to the required cell density, according to the different incubation time points described. 90 μL per well were dispensed in final assay plates Nunclon 96-well flat bottom Solid White and 10 µL of intermediate plates were added to each well, as described before. 4 sets of assay plates were arranged to assay in order to be sequentially stopped at each indicated time point. Top and bottom rows were dismissed for compound assay, to reduce evaporation effects.

Plates were incubated at 37 ºC and 5% CO_2_ for the indicated time points; incubation was stopped by addition of 10 μL of prewarmed Cell Titer Glo reagent (Promega®), and after shaking the plates were incubated at room temperature for 10 min, to allow the signal to settle. Plate luminescence was read on an Infinite F200 plate reader (Tecan), and raw data were processed and analyzed as previously described.

*Reversibility assays*

Mid-log *T. brucei brucei* cultures were adjusted to a working density of 3,500 cell/mL. 90 μL per well were dispensed in final assay plates 96-well transparent Nunclon plates, and 10 µL of intermediate plates were added to each well, as described before. Top and bottom rows were dismissed for compound assay, to reduce evaporation effects.

Plates were incubated at 37 ºC and 5% CO_2_ for 18 h; once drug exposure was finished, plates were spun for 5 min at 500 rpm and room temperature to allow cells to settle at the well bottoms. 95 μL of media was replaced with fresh prewarmed media, and the process was repeated 3 times. Finally, 10 μL of each well were seeded in a new microtiter plate with 90 μL of fresh prewarmed media, on duplicate. Plates were incubated for 72 h, and viability was determined by resazurin reduction as previously described.

*Cytotoxicity assay in MRC5*

Intermediate plates were made as described, adding 95 μL of DMEM complete media to 5 μL of compound per well setting a 5% DMSO amount.

Log-phase MRC5 cells were removed from a T-75 TC flask using TrypLE® Express (Thermo®) and dispersed by gentle pipetting. Cell density was adjusted to working concentration in prewarmed DMEM medium: 25,000 cells in 90 μL of culture were plated in 96-well transparent Nunclon plates and let to settle for 24 h at 37 ºC and 5% CO_2_. After settling incubation, 10 μL of fresh made intermediate plate were added per well: final maximal concentration for compounds was 50 μM in 0.5% DMSO per well. Plates were incubated for 48 h at 37 ºC and 5% CO_2_. 4 h prior to fluorescence measurement, 20 μL of 500 μM resazurin solution was added. Fluorescence was read in an Infinite F200 plate reader (Tecan®) at 550 nm (excitation filter) and 590 nm (emission filter).

A 4-parameter equation was used to fit the dose-response curves and determination of EC_50_ by SigmaPlot ® 13.0 software. Assays were performed in duplicate at least twice for positive compounds, to achieve a minimal n=3 per dose response.

*β-D-Galactosidase Transgenic* T. cruzi *Assay*

A Thermo Scientific Multidrop Combi dispenser (MTX Lab Systems, Vienna, VA) was used to dispense 90 μL of *T. cruzi* amastigote–infected L6 cell culture (4×10^3^ infected L6 cells per well) into 96-well Corning assay plates (Corning Inc., Corning, NY) already containing 10 μL of the compounds to be screened and controls. The plates were incubated at 37 °C for 96 h. Then, 30 μL of 100 μM CPRG and 0.1% NP40 diluted in PBS were added to each well, and the plates were incubated for 4 h at 37 °C in the dark. Absorbance at 585 nm was measured in a Vmax kinetic microplate reader (Molecular Probes). Compound activities were normalized using the in-plate negative (benznidazole at 10 μg/mL) and positive (0.2% DMSO) growth controls.

*Resazurin-Based L6 Assay*

One hundred microliters (100 μL) per well of culture medium containing the compounds and controls were added to L6 cells previously cultured (4×10^3^ L6 cells per well). After 72 h at 37 °C the medium was exchanged and the viable cell number was determined by resazurin (Sigma–Aldrich) reduction. 20 *μ*l of resazurin (1.1 mg/ml) was added to each well and incubated in the dark for 2 h at 37 ***º***C. Cell viability was estimated by measuring the final fluorescence at 570-590 nm in an Infinite F200 plate reader (Tecan).

*Cytotoxicity assay in THP-1*

Cellular toxicity of all compounds was determined using the colorimetric MTT-based assay after incubation at 37 °C for 72 h in the presence of increasing concentrations of compounds (final maximal concentration was 50 μM in 0.5% DMSO per well)[[5](#_ENREF_5)]. The results are expressed as EC_50_ values, the concentration of compound that reduces cell growth by 50% versus untreated control cells. Assays were performed in duplicate at least twice to achieve a minimal n=3 per dose response.

*Determination of EC_50_ in* L. donovani

Macrophage-differentiated THP-1 cells were infected at a macrophage/parasite ratio of 1/10 with stationary *L. donovani* promastigotes for 24 h at 35 °C and 5% CO_2_, and extracellular parasites were removed by washing with PBS. Infected cell cultures were then incubated with different compounds concentrations at 37 °C for 72 h. Luminescence was measured using the Promega kit luciferase assay system (Promega, Madison, WI). Assays were performed in duplicate at least twice, to achieve a minimal n=3 per dose response.

*Assays with* S. mansoni

Somules (40 units/well/96w round-bottomed plate) or adults (five paris/well/24w plate) were cultured in the presence of compounds at 10 µM and phenotypic changes observed as a function of time using an Zeiss Axiovert A1 inverted microscope (Table S4). A constrained nomenclature was used to describe the effects of compounds on parasites (changes in shape, motility and density) [[2](#_ENREF_2), [6](#_ENREF_6)]. To generate a partially quantitative output for comparing drug effects, each descriptor was awarded a score of 1 and these were summed to a maximum score of 4 [[7-10](#_ENREF_7)]: evidence of degeneracy or death was awarded the maximum score of 4. For adults specifically, damage to the tegument (outer surface) was also awarded a score of 4 based on the indication that damage to the patrasite’s surface is lethal *in vivo* [[11](#_ENREF_11)]. Assays were performed twice and representative data are shown.

**References**

1. Garcia-Hernandez R, Gomez-Perez V, Castanys S, Gamarro F. Fitness of *Leishmania donovani* parasites resistant to drug combinations. PLoS Negl Trop Dis. 2015;9:e00037004.

2. Abdulla MH, Ruelas DS, Wolff B, Snedecor J, Lim KC, Xu F, et al. Drug discovery for schistosomiasis: hit and lead compounds identified in a library of known drugs by medium-throughput phenotypic screening. PLoS Negl Trop Dis. 2009;3(7):e478. doi: 10.1371/journal.pntd.0000478. PubMed PMID: 19597541; PubMed Central PMCID: PMCPMC2702839.

3. Duvall RH, DeWitt WB. An improved perfusion technique for recovering adult schistosomes from laboratory animals. Am J Trop Med Hyg. 1967;16(4):483-6. PubMed PMID: 4952149.

4. Colley DG, Wikel SK. Schistosoma mansoni: simplified method for the production of schistosomules. Exp Parasitol. 1974;35(1):44-51. PubMed PMID: 4815018.

5. Gomez-Perez V, Manzano JI, Garcia-Hernandez R, Castanys S, Campos Rosa JM, Gamarro F. 4-Amino bis-pyridinium derivatives as novel antileishmanial agents. Antimicrob Agents Chemother. 2014;58(7):4103-12.

6. Glaser J, Schurigt U, Suzuki BM, Caffrey CR, Holzgrabe U. Anti-Schistosomal Activity of Cinnamic Acid Esters: Eugenyl and Thymyl Cinnamate Induce Cytoplasmic Vacuoles and Death in Schistosomula of Schistosoma mansoni. Molecules. 2015;20(6):10873-83. doi: 10.3390/molecules200610873. PubMed PMID: 26076109.

7. Fonseca NC, da Cruz LF, da Silva Villela F, do Nascimento Pereira GA, de Siqueira-Neto JL, Kellar D, et al. Synthesis of a sugar-based thiosemicarbazone series and structure-activity relationship versus the parasite cysteine proteases rhodesain, cruzain, and Schistosoma mansoni cathepsin B1. Antimicrob Agents Chemother. 2015;59(5):2666-77. doi: 10.1128/AAC.04601-14. PubMed PMID: 25712353; PubMed Central PMCID: PMCPMC4394791.

8. Long T, Neitz RJ, Beasley R, Kalyanaraman C, Suzuki BM, Jacobson MP, et al. Structure-Bioactivity Relationship for Benzimidazole Thiophene Inhibitors of Polo-Like Kinase 1 (PLK1), a Potential Drug Target in Schistosoma mansoni. PLoS Negl Trop Dis. 2016;10(1):e0004356. doi: 10.1371/journal.pntd.0004356. PubMed PMID: 26751972; PubMed Central PMCID: PMCPMC4709140.

9. Long T, Rojo-Arreola L, Shi D, El-Sakkary N, Jarnagin K, Rock F, et al. Phenotypic, chemical and functional characterization of cyclic nucleotide phosphodiesterase 4 (PDE4) as a potential anthelmintic drug target. PLoS Negl Trop Dis. 2017;11(7):e0005680. doi: 10.1371/journal.pntd.0005680. PubMed PMID: 28704396; PubMed Central PMCID: PMCPMC5526615.

10. Weeks JC, Roberts WM, Leasure C, Suzuki BM, Robinson KJ, Currey H, et al. Sertraline, paroxetine, and chlorpromazine are rapidly acting antherlmintic drugs capable of clinical repurposing. Scientific Reports. 2018;8. doi: 10.1038/s41598-017-18457-w.

11. Andrews P, Thomas H, Pohlke R, Seubert J. Praziquantel. Med Res Rev. 1983;3(2):147-200. PubMed PMID: 6408323.
