# Supplementary material for: Evaluation of a class of isatinoids identified from a high-throughput screen of human kinase inhibitors as anti-Sleeping Sickness agents
Source: PLoS Negl Trop Dis. 2019 Feb 8;13(2):e0007129. doi: 10.1371/journal.pntd.0007129 (PMC6383948; doi:10.1371/journal.pntd.0007129)
Supplement: S3 Text — (DOCX) [file pntd.0007129.s010.docx]

**S3 Text.** ADME experiment protocols.

*Aqueous pH 7.4 Solubility*

Compounds are dried down from 10 mM DMSO solutions using centrifugal evaporation technique. Phosphate buffer (0.1 M pH 7.4) added and StirStix inserted in the glass vials, shaking is then performed at a constant temperature of 25 °C for 20-24 h. This step is followed by double centrifugation with a tip wash in between, to ensure that no residues of the dried compound are interfering. The solutions are diluted before analysis and quantification using LC/MS/MS is performed.

*Log D_7.4_*

Shake-flask octanol-water distribution coefficient at pH 7.4 (Log *D*_7.4_). The aqueous solution used is 10 mM sodium phosphate pH 7.4 buffer. The method has been validated for Log *D*_7.4_ ranging from -2 to 5.0.

*Human Plasma Protein Binding (PPB)*

PPB is determined using equilibrium dialysis (RED device) to separate free from bound compound.  The amount of compound in plasma (10 µM initial concentration) and in dialysis buffer (pH 7.4 phosphate buffer) is measured by LC-MS/MS after equilibration at 37 °C in a dialysis chamber. The fraction unbound (fu) is reported.

*Human Liver Microsomal CL_int_*

*In vitro* intrinsic clearance determined from human liver microsomes using a standard approach[[12](#_ENREF_12)]. Following incubation and preparation, the samples are analyzed using LC/MS/MS. Refined data are uploaded to IBIS and are displayed as CL_int_ (intrinsic clearance) in μl/min/mg.

*Rat Hepatocyte CL_int_*

*In vitro* intrinsic clearance determined from rat hepatocytes using a standard approach[[12](#_ENREF_12)]. Following incubation and preparation, the samples are analyzed using LC/MS/MS. Refined data are uploaded to IBIS and are displayed as CL_int_ (intrinsic clearance) μl/min/1 million cells.

**References**

1. Konsoula R, Jung M. *In vitro* plasma stability, permeability, and solubility of mercaptoacetamide histone deacetylase inhibitors. International Journal of Pharmaceutics. 2008;361:19-25.
